# E-learning readiness among dental students and faculty: a comparative study before and after the COVID-19 pandemic

**DOI:** 10.3389/fmed.2024.1306205

**Published:** 2024-05-30

**Authors:** Talal M. Zahid, Shoroog Agou

**Affiliations:** ^1^Department of Periodontology, Faculty of Dentistry, King Abdulaziz University, Jeddah, Saudi Arabia; ^2^Department of Orthodontics, Faculty of Dentistry, King Abdulaziz University, Jeddah, Saudi Arabia

**Keywords:** dental education, e-learning, COVID-19, e-learning preferences post pandemic, online learning, e-learning readiness

## Abstract

**Background:**

The COVID-19 pandemic accelerated the global adoption of e-learning, even in institutions that had previous reservations. Nevertheless, the impact of this transformation on dental education remains unclear. This study aimed to assess the e-learning readiness of dental students and faculty before and after COVID-19. It also explored their post-pandemic e-learning preferences for dental education.

**Methods:**

Cross-sectional surveys were conducted at King Abdulaziz University’s Faculty of Dentistry (KAUFD) in Jeddah, Saudi Arabia both before and after COVID-19. Faculty and students from two distinct cohorts were recruited at two time points. Participants completed a detailed questionnaire on e-learning readiness across multiple domains. Statistical analysis was performed using R v 3.6.3. Descriptive and group comparisons were conducted using chi-squared test, unpaired *t*-test, and Spearman’s correlation.

**Results:**

1,057 responses (response rate = 99.8%) were analyzed: 2015 (*n* = 400) and 2021 (*n* = 657). Both faculty and students demonstrated significant improvements in e-learning readiness across all domains from 2015 to 2021. In 2021, faculty members scored significantly higher than students in almost all readiness domains, except for e-learning experience (*p* < 0.001 for all domains). After the pandemic, both groups preferred a blended learning model: 75% traditional and 25% online education. A significant increase in typing and editing training requests by faculty and students was observed in 2021. Students showed a decline in training needs for web and online tool usage.

**Conclusion:**

The COVID-19 pandemic pushed the rapid adoption of e-learning in dental education. In this study, faculty showed greater e-learning readiness, but students voiced concerns about missed in-person interactions, social isolation, and screen fatigue. Further multi-institutional studies are required for more comprehensive insights.

## Introduction

E-learning is learning utilizing formalized teachings but with the help of digital tools, typically via the internet. E-learning readiness, on the other hand, refers to the preparedness of an institution, organization, or a person to engage in e-learning activities (1). E-learning has evolved as an effective method of education for distance learners over the last 20 years (2, 3). While ongoing research is being conducted on the application of e-learning in educational settings, studies published to date suggest its certain advantages over conventional educational methods (2–7). The noted advantages of using e-learning include lower cost, more flexibility and versatility, and easier access. In fact, e-learning can address some of the common problems in the traditional education system. These may include faculty member shortages, limited resource accessibility, and inadequate classroom space (2, 3, 5). Moreover, a growing body of studies now suggests e-learning as an inclusive mode of education that caters to various learning styles (5–9).

After the COVID-19 outbreak in 2019, e-learning overnight became the primary mode of education for millions of students across the globe. Higher education institutions were forced to adopt digital platforms and transition to online learning at an unprecedented scale (9). This abrupt shift from onsite to remote education occurred even though the efficacy of online teaching and learning had not been thoroughly studied under similar circumstances (4, 6, 8–10). As a result, despite insufficient evidence, universities and colleges worldwide incorporated e-learning into their curricula to continue teaching activities. This sudden change has actually accelerated the adoption of e-learning approaches, overcoming previous resistances (4, 9, 10). However, the effects of this change on dental school students and faculty remain uncertain. Hence, there is a need to assess e-learning success factors to create effective online learning methods and maintain such advancements in the future.

In recent years, the Kingdom of Saudi Arabia has taken numerous initiatives to implement sustainable e-learning strategies, including in the field of dental education. The nation has acknowledged the significance of implementing top-notch educational practices in line with the demands of globalization to meet upcoming challenges (9, 11, 12). Accordingly, the Faculty of Dentistry at King Abdulaziz University (KAUFD) incorporated e-learning strategies in its curriculum to raise the standard of online teaching and learning. This incorporation, however, happened long before the COVID-19 pandemic hit the world (8, 13).

Before the pandemic, a study conducted at KAUFD assessed the readiness of students and faculty for online dental education. It revealed that while students exhibited acceptable levels of personal traits and system competency, their readiness for e-learning decreased with age (12). However, it should be noted that KAUFD introduced its first structured e-learning program only after the pandemic hit. A study was conducted afterward to measure the perceptions and overall experiences of dental students using the structured program (8). The results indicated that students had a favorable view of e-learning in terms of its value, usability, and opportunity for self-reflection. However, the study was subjected to biases due to its use of a convenience sampling technique and a low response rate, which limits the generalizability of the results. Hence, there is a need for more comprehensive studies to effectively evaluate e-learning necessities in dental education.

Therefore, the aim of this study was to investigate the readiness of dental students and faculty members at KAUFD toward e-learning, specifically online teaching and learning, both before and after the COVID-19 pandemic. Additionally, we aimed to explore post-pandemic e-learning preferences in order to plan for a meaningful and effective integration of e-learning modalities in dental education.

## Materials and methods

### Research design

A comparative cross-sectional survey was conducted at KAUFD in Jeddah, Saudi Arabia to explore the readiness of dental students and faculty to adopt e-learning for online teaching and learning. This study involved two time points, at each time point, students studying at KAUFD were evaluated. Hence, the two time points represent two cohorts of students. The Institutional Review Board at KAUFD reviewed and approved this study. Informed consent was obtained from all participants before the study began.

### Sample selection

We approached two distinct cohorts of faculty and students attending KAUFD at two different time points: before the onset of the COVID-19 pandemic (in 2015) and in the post-pandemic era (in 2022). Initially, we aimed for a total sample approach, intending to include all members of these cohorts. However, our sampling method aligned more closely with convenience sampling due to practical constraints. We recruited all subjects who agreed to participate. This approach provided a representative snapshot of the attitudes and experiences of participants within our institution during these periods.

### Data collection

We created the questionnaire using Google Forms and distributed it to students and faculty via email. Follow-up reminders were sent 2 weeks and 1 month after the initial email. Participants were asked to complete the questionnaire by scanning the QR code with their smartphones or tablets. This same recruitment process was employed for cohorts of both time points.

### Questionnaire design

The primary objective of this study was to explore the readiness of dental students and faculty to adopt e-learning for online teaching and learning. The study used a theoretical model, similar to the ones proposed and validated by Al-Harbi (14, 15) and Cidral et al. (16) that included constructs and relationships of factors that influence e-learning readiness. These constructs are categorized into four basic domains: (1) individual characteristics, (2) system competency requirements, (3) social influence, and (4) institutional support. Linjawi et al. (12) also adopted this model in a similar population.

Based on the questionnaire model developed by Al-Harbi (14, 15), we adopted their questionnaire to assess the readiness of students and faculty members on e-learning and carefully fitted it to the unique requirements of our current investigation while also adding elements from earlier research (12, 16). The questionnaire was reviewed by two doctorate of philosophy (PhD) degree holder with extensive experience in biostatistics. The questionnaire had a total of 36 questions: 31 questions on e-learning readiness using a five-point Likert scale (17), one question on experience, one question addressing challenges, one question regarding needed support, and two global questions. The domains were designed as follows.

#### Individual characteristics domain

- *Demographics*: This subdomain included questions on age, gender, and grade. For faculty members, four additional questions were asked, including the subject they teach, their degree and the university they graduated from, the location and year it was obtained, and years of teaching experience.
- *Computer skills*: This subdomain designed to evaluate the participants’ computer proficiency. Topics covered in the questions included operating a computer, installing or setting up software, writing and editing using Office applications, formatting documents, managing multimedia files, and ability to multitask with multiple open windows.
- *E-learning experience*: This subdomain assessed participants’ experience and participation in e-learning activities, such as online courses, discussions, exams, and workshops.

#### System competency needs domain (multiple Likert scale-based questions)

- *Perceived ease of use*: this subdomain analyzed participants’ perceptions of online tool usage for personal and educational purposes. It consisted of the following:
  - *Online skills:* assessed participants’ perceived abilities in using various online services and tools such as searching information on the internet, sending and receiving emails, downloading and uploading files, and engaging with social media platforms.
  - *Motivation level:* evaluated participants’ perceived motivation when tackling extensive online tasks independently, such as staying focused during online lectures, completing online tasks despite distractions, dedicating 10–20 h per week to online lectures, and setting specific goals and deadlines.
  - *English literacy:* assessed participants’ perceived level of proficiency in online communication, particularly in overcoming language barriers.
- *Perceived usefulness*: this subdomain investigated the following two variables:
  - *Importance of online technology* for success in both personal and educational contexts.
  - *Impact of e-learning on dental education.*
- *Technology accessibility*: this subdomain evaluated the availability of all necessary technological resources for e-learning implementation, such as hardware, software, internet connectivity, and mobile devices.

#### Social influence domain or social norms

Social influence domain or social norms assessed the influence of surrounding people (e.g., friends, family members, and educators) on participants’ perception and use of online services.

#### Institutional support domain

Institutional support domain analyzed the significance of technical and administrative institutional support for the successful adoption of e-learning in dental education.

#### Overall readiness domain

Overall readiness domain evaluated participants’ overall readiness for adopting e-learning in dental education.

#### Needed technical support domain

Needed technical support domain assessed whether the participants need technical support to adopt e-learning strategies in dental education.

### Open-ended questions

There were also two open-ended questions at the end of the questionnaire. The first question was designed to explore participants’ thoughts on potential challenges and concerns in adopting e-learning in dental education. The second question sought solutions and recommendations for overcoming these challenges.

### Supplemental questionnaire

The same questionnaire was distributed in 2015 and 2021. In the 2015 questionnaire, however, questions related to English literacy, perceived usefulness, social influence, and institutional support were excluded for students, as they were considered more pertinent to faculty members. In contrast, both groups received the same set of questions in the 2021 questionnaire.

The 2021 questionnaire also included a supplemental section, which comprised a total of eight questions. Four questions explored the pandemic’s impact on e-learning using a five-point Likert scale; one question addressed post-pandemic preferences; one assessed learning quality; and two open-ended questions focused on challenges and solutions. Specific topics covered included opinions on the effects of COVID-19 on readiness, the online learning environment, study plans and learning goals, encountered difficulties, time management, and overall learning experience. Participants were also asked about their transition back to regular life activities and the quality of online learning experiences in the university. The appendix section includes a detailed questionnaire, along with the individual questions.

### Statistical analysis

Statistical Analysis was performed using R v 3.6.3. Counts and percentages were used to summarize categorical variables. The mean ± standard deviation (SD) and the median/interquartile range (IQR) were used for continuous normal and non-normal variables, respectively. Unpaired *t*-test was used to compare continuous variables between groups, and the Chi-square test was used for categorical variables. Spearman’s correlation was used to assess the association between continuous variables. Hypothesis testing was performed at 5% level of significance. The percentage (%) of readiness was calculated by dividing the readiness score by the maximum possible score.

## Results

The total response rate was 99.8% (*n* = 1059/1061). Two questionnaires were excluded due to missing major data, representing only 0.1% of responses. A total of 1,057 responses were included; of these, 400 responses were from 2015 (faculty members, *n* = 50; students, *n* = 350), while 657 responses were from 2021 (faculty members, *n* = 201; students, *n* = 456).

### Sample characteristics

The mean age of faculty members was 42 years in 2015 and 40 years in 2021. The mean age of students was 24 years in 2015 and 22 years in 2021 (Table 1). There was no significant difference in gender distribution between the two groups, with a *p* value of 0.071.

**Table 1:** Demographics of the study sample (stratified by year and group).

		Faculty 2015 (*n* = 50)	Faculty 2021 (*n* = 201)	Student 2015 (*n* = 350)	Student 2021 (*n* = 456)
Age	Mean (SD)	42.5 (11.6)	39.7 (9.02)	24.2 (2.85)	22.3 (1.57)
Gender					
*Male*	*n* (%)	28 (56.0%)	100 (49.8%)	202 (57.7%)	223 (48.9%)
*Female*	*n* (%)	22 (44.0%)	101 (50.2%)	148 (42.3%)	233 (51.1%)

SD, Standard deviation.

### Assessment of e-learning readiness across domains

Results showed that both faculty and students experienced statistically significant improvements in almost all domains of e-learning readiness between 2015 and 2021 (Table 2). In the post-COVID-19 period, both faculty and students demonstrated significant improvements in technological access, with faculty’s mean score increasing from 4.36 to 4.65 (*p* < 0.001) and students’ mean score from 4.04 to 4.21 (*p* < 0.001). This indicates better technological access for both groups in 2021 compared to 2015. A similar pattern was noted in computer skills, online skills, motivation level, and overall readiness variables. However, unlike the results observed in faculty members for the computer skills subdomain, no significant difference was seen in students between pre-and post-COVID-19 periods (*p* = 0.122).

**Table 2:** Distribution of scores for the study constructs across students and faculty members (stratified by year).

Domain	Variables	Faculty 2015^a^ *Mean (SD)*	Faculty 2021^b^ *Mean (SD)*	Students 2015^c^ *Mean (SD)*	Students 2021^d^ *Mean (SD)*	*p* (F2015 vs. F2021)	*p* (F2015 vs. S2015)	*p* (F2021 vs. S2021)	*p* (S2015 vs. S2021)	Faculty overall *(n = 251)*	Student overall *(n = 806)*	*p* (overall)
Individual characteristics domain	Computer skills	4.25 (0.64)	4.46 (0.63)	4.07 (0.66)	4.10 (0.80)	0.015^*ab^	0.093	<0.001^*bd^	0.122	4.42 (0.63)	4.09 (0.74)	<0.001^*^
	E-learning experience	2.86 (1.26)	2.67 (1.49)	3.81 (0.66)	3.51 (0.73)	0.784	<0.001^*ac^	<0.001^*bd^	<0.001^*cd^	2.71 (1.45)	3.64 (0.71)	<0.001^*^
System competency domain	*Perceived ease of use*											
	Online skills	3.97 (0.83)	4.58 (0.39)	4.00 (0.80)	4.21 (0.74)	<0.001^*ab^	0.816	<0.001^*bd^	<0.001^*cd^	4.46 (0.56)	4.12 (0.77)	<0.001^*^
	Motivation level	3.82 (0.72)	4.52 (0.47)	3.52 (0.77)	3.87 (0.82)	<0.001^*ab^	0.004^*ac^	<0.001^*bd^	<0.001^*cd^	4.38 (0.60)	3.72 (0.82)	<0.001^*^
	English literacy	4.56 (1.05)	4.59 (0.51)	NA	4.12 (1.06)	0.048^*ab^	NA	<0.001^*bd^	NA	4.58 (0.65)	4.12 (1.06)	<0.001^*^
	*Perceived usefulness*											
	Perceived usefulness—importance	4.56 (0.64)	4.66 (0.49)	NA	4.33 (0.77)	0.480	NA	<0.001^*bd^	NA	4.64 (0.52)	4.33 (0.77)	<0.001^*^
	Perceived usefulness—impact	4.26 (0.56)	4.38 (0.74)	4.04 (0.85)	3.76 (1.12)	0.077	0.166	<0.001^*bd^	0.007^*cd^	4.36 (0.71)	3.88 (1.02)	<0.001^*^
	*Technology accessibility*											
	Technological access	4.36 (0.55)	4.65 (0.36)	4.04 (0.69)	4.21 (0.63)	<0.001^*ab^	0.001^*ac^	<0.001^*bd^	<0.001^*cd^	4.59 (0.42)	4.14 (0.67)	<0.001^*^
Social influence	Social influence	3.99 (0.98)	4.51 (0.48)	NA	4.01 (0.86)	0.001^*ab^	NA	<0.001^*bd^	NA	4.41 (0.65)	4.01 (0.86)	<0.001^*^
Institutional support	Institutional support	4.56 (0.81)	4.55 (0.51)	NA	4.14 (0.93)	0.193	NA	< 0.001^*bd^	NA	4.55 (0.58)	4.14 (0.93)	<0.001^*^
Overall readiness	Overall readiness (%)	78.0 (16.8)	88.9 (14.5)	69.2 (21.3)	75.9 (21.1)	<0.001^*ab^	0.005^*ac^	<0.001^*bd^	<0.001^*cd^	--	--	--

NA, Not applicable. Analysis was performed using one-way ANOVA for the overall association and using unpaired *t*-test with Tukey’s adjustment for pairwise comparisons.^*^p < 0.05 indicates statistical significance, symbols a, b, c, and d represent the mean scores for Faculty 2015, Faculty 2021, Students 2015, and Students 2021, respectively. Combined symbols (e.g., ab) indicate a significant difference between the groups (e.g., Faculty 2015 vs. Faculty 2021).

Several variables remained relatively stable for faculty members, including English literacy (from 4.56 to 4.59, *p* = 0.048), institutional support (from 4.56 to 4.55, *p* = 0.193), and perceived usefulness in terms of importance (from 4.56 to 4.66, *p* = 0.480). A decline was observed in e-learning experience after the pandemic for both faculty members (from 2.86 to 2.67, *p* = 0.784) and students (from 3.81 to 3.51, *p* < 0.001).

### Faculty members vs. students

The mean score for all domains was significantly higher in faculty members than in students, except for the e-learning experience score, which was higher in students. All comparisons were statistically significant at the 0.001 level (Table 2).

### Overall readiness and perception

The analysis of 2021 supplemental questionnaire revealed a significant difference between students and faculty members in terms of preparedness for online learning, with faculty members showing a higher level of readiness (*p* < 0.001) (Table 2). Among faculty members, two-thirds strongly agreed that the pandemic had improved their online teaching readiness, while only one-quarter of the students felt the same way (*p* < 0.001) (Table 3). When asked about their preferences if normal life activities were to resume, the majority of both groups still favored a mix of traditional and online education (*p* = 0.001). The largest percentage of students (27.4%) and the majority of faculty (39.3%) preferred a mix of 75% traditional and 25% online education. Faculty members also rated the quality of the online learning experience significantly higher than students did (*p* < 0.001).

**Table 3:** Overall readiness and perception toward online teaching and learning in 2021.

	Faculty	Student	*p* (overall)
	*n* = 201	*n* = 453	
Overall, I think I was well-prepared for online teaching/learning:			<0.001^*^
Strongly disagree	1 (0.50%)	41 (9.05%)	
Disagree	6 (2.99%)	54 (11.9%)	
Neutral	9 (4.48%)	104 (23.0%)	
Agree	91 (45.3%)	143 (31.6%)	
Strongly agree	94 (46.8%)	111 (24.5%)	
I believe the COVID-19 pandemic experience has improved my online teaching/learning readiness:			<0.001^*^
Strongly disagree	0 (0.00%)	38 (8.39%)	
Disagree	0 (0.00%)	53 (11.7%)	
Neutral	9 (4.48%)	98 (21.6%)	
Agree	59 (29.4%)	145 (32.0%)	
Strongly agree	133 (66.2%)	119 (26.3%)	
If we were to go back to normal life activities, I would prefer to:			0.001^*^
Traditional face-to-face 100%	22 (10.9%)	66 (14.6%)	
Traditional face-to-face 75%	79 (39.3%)	124 (27.4%)	
Traditional face-to-face 50%	29 (14.4%)	123 (27.2%)	
Traditional face-to-face 25%	60 (29.9%)	114 (25.2%)	
Online learning 100%	11 (5.47%)	26 (5.74%)	
Overall perception regarding the quality of online learning experience	9.00 [9.00;10.0]	8.00 [7.00;9.00]	<0.001^*^

Counts and percentages were used to summarize categorical data, and the median [IQR] was used to summarize continuous data. Continuous data were compared using the Mann–Whitney test, while categorical data were compared using the Chi-square test of independence. A *p* value of less than 0.05 was considered statistically significant. The asterisk () indicates a *p* value of less than 0.05.

### Extra training requests

**Figure 1:**
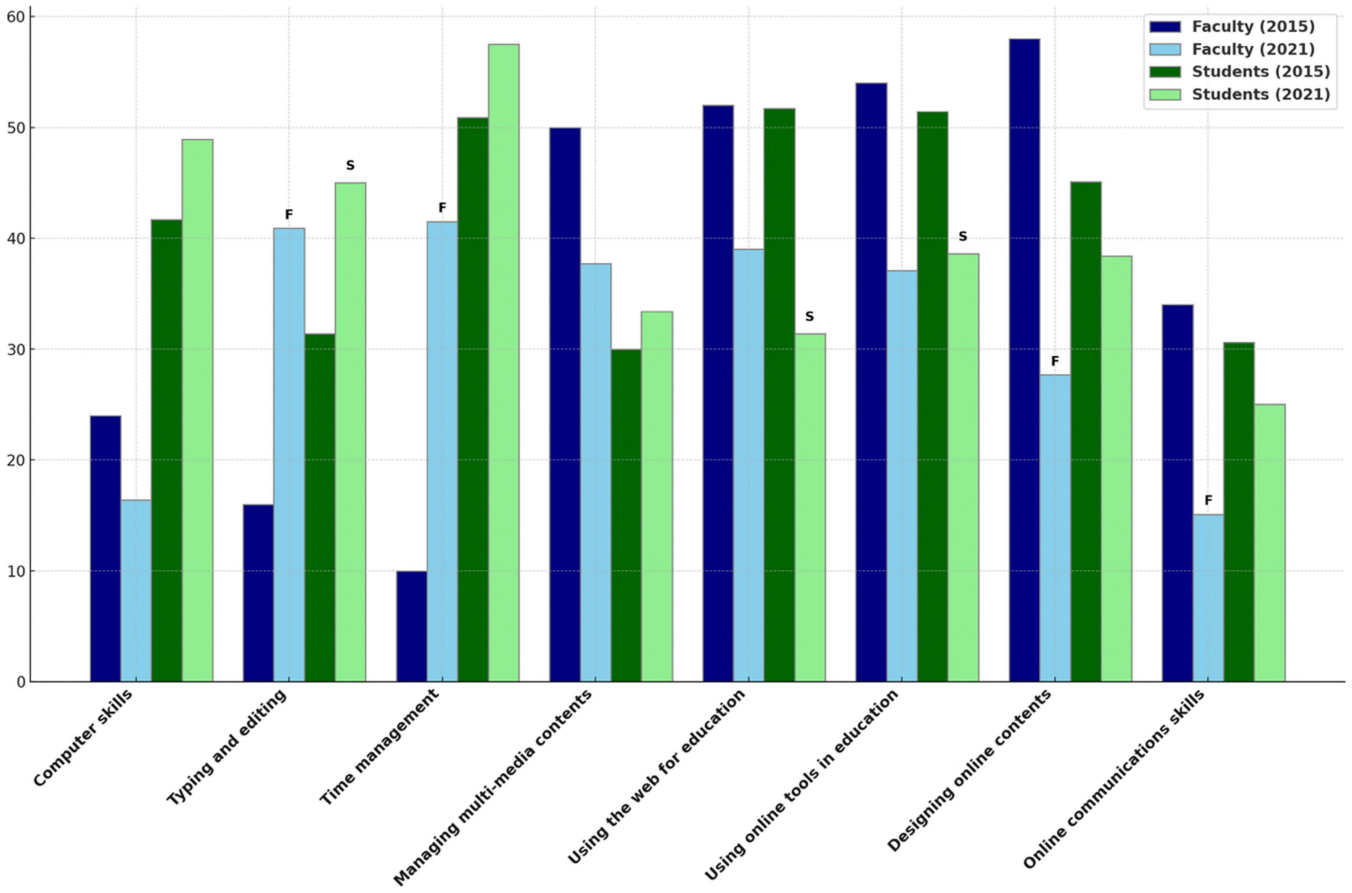
Comparison of training needs in technology use: Faculty vs. Students (2015 vs. 2021). The graph illustrates the percentage of faculty and students who reported a need for additional training in various technology use categories in 2015 and 2021. Symbols “F” and “S” above the bars represent statistically significant changes over the years for faculty and students, respectively, with a *p* value less than 0.05 indicating a significant difference in reported training needs between the 2 years.

Between 2015 and 2021, faculty members experienced a significant increase in requests for training in typing and editing (from 16.0 to 40.9%, *p* = 0.002) and time management (from 10.0 to 41.5%, *p* < 0.001), while demand for designing online content significantly decreased (from 58.0 to 27.7%, *p* < 0.001) (Figure 1). For students, a significant increase in requests for typing and editing training was observed (from 31.4 to 45.0%, *p* < 0.001), whereas the need for training in using the web for education (from 51.7 to 31.4%, *p* < 0.001) and using online tools in education (from 51.4 to 38.6%, *p* < 0.001) significantly declined during the same period.

## Discussion

The COVID-19 pandemic has caused a paradigm shift in education. It forced both teachers and students to move from in-person classrooms to digital platforms. They had a totally different experience due to this shift in learning approach. The present study aimed to investigate the readiness of faculty and students toward online teaching and learning both before and after the COVID-19 pandemic. The study also explored post-pandemic preferences of both groups to effectively incorporate e-learning into dental education.

The results of this study revealed significant improvements in various domains of e-learning readiness between 2015 and 2021 among both faculty members and students. In the post-pandemic period, there was a notable increase in the overall readiness score for faculty members, indicating a positive trend in the adoption of online teaching and learning methods. Similarly, students’ overall readiness also improved during the same period. These results could be attributed to the extensive use of online learning tools and platforms due to the COVID-19 pandemic, which forced educational institutions to adapt to remote learning methods (4, 6, 8).

In this study, however, we also observed significant differences in the readiness and perception scores between faculty and students. Faculty members had higher scores in almost all domains than students. They also rated their quality of online learning experience higher than students. The only subdomain in which students had an edge was e-learning activities and involvement. It is possible that faculty members either found it easier or were under more pressure to adapt to the remote learning environment than students due to their responsibility to teach and role as content creators (18, 19). In fact, they also had an increased exposure to online teaching tools and resources during the pandemic (10, 13, 18).

In contrast, we observed that students were comparatively less engaged with e-learning than faculty. Indeed, in their responses to open-ended questions, students explicitly mentioned several challenges as barriers to fully adapting to the e-learning environment, including lack of in-person interactions, difficulty communicating with instructors, social media distractions, prolonged screen time, technical difficulties, and time management. One student actually reported that his ability to grasp concepts during e-learning session is significantly reduced compared to face-to-face instruction. These concerns align with previous studies that have identified similar challenges faced by students in e-learning environments (20–22). In fact, prior literature has shown that prolonged engagement with online platforms can lead to e-learning fatigue, which affects both faculty and students (22–26). Taken together, the presence of these barriers might contribute to students feeling less engaged, leading to a lesser perceived quality of online learning.

Furthermore, Vygotsky’s social constructivist theory emphasizes that learning is fundamentally a social process, deeply influenced by interaction within a cultural context (27, 28).

This perspective is particularly relevant as students experienced significant social isolation during the lockdown, impacting their mental well-being and potentially reducing their motivation to engage with e-learning platforms (21, 22, 24, 29). Such isolation likely contributed to students’ expressed preferences for learning models that are more interactive and socially engaging. It underscores the critical need for e-learning platforms to address not only the academic requirements of students but also their psychological well-being (29). In our study, students suggested effective scheduling and opening the cameras as solutions to deal with these barriers.

Taken together, it can be said that the COVID-19 pandemic brought several lessons to the forefront for dental schools. Dental schools can use e-learning to provide students with access to a broader range of learning resources, including virtual simulations, online textbooks, and interactive multimedia content (2, 3). Additionally, e-learning can help to overcome some of the challenges associated with traditional teaching methods, such as limited access to clinical training and the high cost of materials (5–9). However, there is also a need to consider the limitations of e-learning. Given students’ difficulties with feelings of isolation and time management, dental schools need to work on developing a more engaging and interactive online learning environment that rationally blends both online and face-to-face instructions. Incorporating elements like group discussions, peer interactions, and virtual patient cases might capture the collaborative spirit of in-person learning (4, 10, 16). Moreover, adopting a flipped classroom design, where students acquire the didactic part before class, and face-to-face sessions are saved for higher order thinking and psychomotor skills might be a better model for dental schools to adopt.

The majority of students and faculty believe the pandemic has significantly improved their e-learning readiness, supporting the general notion that COVID-19 has accelerated the e-learning learning curve. That said, there is plenty of room for institutional support to improve the e-learning experience for faculty and students. In our study, students and faculty requested support in enhancing their skills like time management, typing and editing, using AI tools for education, and general computing skills among many other skills.

### Limitations, recommendations for practice and future studies

This study presents several limitations. Since our research was based on data from a single dental school (i.e., KAUFD), the findings of this study are not generalizable. Another limitation was relying solely on digital platforms to reach out participants. This meant we missed out on the depth and subtlety that face-to-face interviews could bring. We also relied on total sample approach because of time constraints. This choice might have limited the generalizability of the results. Finally, although the Likert scale questions streamlined the responses, this might have restricted the depth of insights that other questioning techniques could have uncovered.

Moving forward, future studies should explore the effects of e-learning on student’s overall performances. By comparing our data with other dental schools and health institutions, a more comprehensive understanding can be gained on this. Dental schools should thus continue investing in e-learning to better evaluate its efficacy and acceptance. Long-term studies evaluating e-learning challenges are needed to reap the full advantages of e-learning. Additionally, to ensure the generalizability of the results, these studies should also include students from various disciplines across Saudi universities.

## Conclusion

The COVID-19 pandemic has accelerated the adoption of e-learning in dental education, revealing both its advantages and drawbacks. While faculty members have showed more readiness to e-learning, students expressed concerns, particularly around social isolation, screen fatigue, and the lack of in-person interactions. Hence, it is important to ensure that e-learning platforms also cater to students’ social and psychological needs. While findings from our single-school study provide valuable insights, a broader examination across multiple institutions is required for a comprehensive understanding. Therefore, dental schools should continue their investment in e-learning to shape a balanced and effective educational future.

## Data availability statement

The raw data supporting the conclusions of this article will be made available by the authors, without undue reservation.

## Ethics statement

The Institutional Review Board at KAUFD reviewed and approved this study (Protocol# 147-12-20). We obtained a written informed consent from all participants before the study began.

## Author contributions

TZ: Data curation, Formal analysis, Methodology, Resources, Software, Visualization, Writing – original draft, Writing – review & editing. SA: Conceptualization, Data curation, Formal analysis, Methodology, Resources, Supervision, Visualization, Writing – original draft, Writing – review & editing.
