# Corrigendum: E-learning readiness among dental students and faculty: a comparative study before and after the COVID-19 pandemic

**DOI:** 10.3389/fmed.2025.1588843

**Published:** 2025-04-11

**Authors:** Talal M. Zahid, Shoroog Agou

**Affiliations:** ^1^Department of Periodontology, Faculty of Dentistry, King Abdulaziz University, Jeddah, Saudi Arabia; ^2^Department of Orthodontics, Faculty of Dentistry, King Abdulaziz University, Jeddah, Saudi Arabia

**Keywords:** dental education, e-learning, COVID-19, e-learning preferences post pandemic, online learning, e-learning readiness

In the published article, there was an error in the **Funding statement**. [The author(s) declare that no financial support was received for the research, authorship, and/or publication of this article.]. The correct Funding statement appears below.

